# SDF-1*α*/OPF/BP Composites Enhance the Migrating and Osteogenic Abilities of Mesenchymal Stem Cells

**DOI:** 10.1155/2021/1938819

**Published:** 2021-08-13

**Authors:** Linli Li, Xifeng Liu, Bipin Gaihre, Sungjo Park, Yong Li, Andre Terzic, Lichun Lu

**Affiliations:** ^1^Department of Physiology and Biomedical Engineering, Mayo Clinic, Rochester, MN 55905, USA; ^2^Department of Orthopedic Surgery, Mayo Clinic, Rochester, MN 55905, USA; ^3^Department of Orthopedics, Shanghai Fifth People's Hospital, Fudan University, Shanghai 200040, China; ^4^Department of Cardiovascular Diseases and Center for Regenerative Medicine, Mayo Clinic, Rochester, MN 55905, USA

## Abstract

*In situ* cell recruitment is a promising regenerative medicine strategy with the purpose of tissue regeneration without stem cell transplantation. This chemotaxis-based strategy is aimed at ensuring a restorative environment through the release of chemokines that promote site-specific migration of healing cell populations. Stromal cell-derived factor-1*α* (SDF-1*α*) is a critical chemokine that can regulate the migration of mesenchymal stem cells (MSCs). Accordingly, here, SDF-1*α*-loaded microporous oligo[poly(ethylene glycol) fumarate]/bis[2-(methacryloyloxy)ethyl] phosphate composites (SDF-1*α*/OPF/BP) were engineered and probed. SDF-1*α*/OPF/BP composites were loaded with escalating SDF-1*α* concentrations, namely, 0 ng/ml, 50 ng/ml, 100 ng/ml, and 200 ng/ml, and were cocultured with MSC. Scratching assay, Transwell assay, and three-dimensional migration model were utilized to assess the migration response of MSCs. Immunofluorescence staining of Runx2 and osteopontin (OPN), ELISA assay of osteocalcin (OCN) and alkaline phosphatase (ALP), and Alizarin Red S staining were conducted to assess the osteogenesis of MSCs. All SDF-1*α*/OPF/BP composites engendered a release of SDF-1*α* (>80%) during the first four days. SDF-1*α* released from the composites significantly promoted migration and osteogenic differentiation of MSCs documented by upregulated expression of osteogenic-related proteins, ALP, Runx2, OCN, and OPN. SDF-1*α* at 100 ng/ml was optimal for enhanced migration and osteogenic proficiency. Thus, designed SDF-1*α*/OPF/BP composites were competent in promoting the homing and osteogenesis of MSCs and thus offer a promising bioactive scaffold candidate for on-demand bone tissue regeneration.

## 1. Introduction

Biological repair of bone defects has been a major challenge for orthopedics [1, 2]. Autologous bone, allogenic bone, or osteoconductive biomaterials have been successfully used for the repair of bone defects in certain practices [3–5]. However, these methods have some limitations, such as donor site morbidity, limited sources, and immune rejection [3–5]. Tissue-engineered bone could be an effective substitute, with excellent bone regeneration potential and feasibility for commercialization [6, 7].

Conventional tissue engineering strategies depend on the transplantation of stem cells into biomaterials to recreate a functional tissue-equivalent [8, 9]. These methods also have several limitations, such as donor site morbidity, immune rejection, and transplanted cell apoptosis due to devascularization [3, 10]. Thus, an alternative method was designed to avoid these limitations, intending to induce the recruitment of stem cells to the scaffolds coated with chemokine at the bone defect sites. This method can recruit endogenous stem cells to the defect site to play reparative roles, maximizing the body's autoreparative ability [11]. Even though a lot of chemokines participate in this process, stromal cell-derived factor 1*α* (SDF-1*α*) has been recognized as one of the key factors [12].

SDF-1 is a 10 kDa chemokine that contains six spliced isoforms, of which SDF-1*α* is the main expressed isoform [12]. Previous studies have shown that SDF-1*α* exerts pleiotropic effects in ischemic heart diseases: the gradient of SDF-1*α* could guide the recruitment of stem cells to the ischemic sites and also play a protective role through the activation of prosurvival signal transduction pathways [13, 14]. Its chemotactic and antiapoptotic properties indicate that this biochemical factor would be an ideal choice for cell homing studies [15].

Recent studies also showed the potential of SDF-1*α* in bone repair because it can stimulate bone marrow stromal stem cell (BMSC) migration and promote bone formation in bone defect models [16–18]. SDF-1*α* can also exert an antiapoptotic effect, protecting immature human BMSCs from apoptosis [1]. Wynn et al. showed that SDF-1*α* at the concentration of 30-50 ng/ml was optimal for the migration of human BMSCs [19]. The application of SDF-1*α* is limited by its short half-life and easy degradation by enzymes [13, 14]. Thus, the design of a suitable scaffold to both maintain the biological activity of SDF-1*α* and deliver it in a controlled manner remains a challenge.

Oligo[poly(ethylene glycol) fumarate] (OPF), a synthetic polymer, holds great potential as a drug delivery vehicle as it is biodegradable and can be developed into injectable formulations [20, 21]. Through the formation of a cross-linked hydrogel, OPF can act as a scaffolding matrix capable of promoting cell adhesion, proliferation, and differentiation, while also degrade in a predictable way [20, 21]. Moreover, incorporation of bis(2-(methacryloyloxy)ethyl) phosphate (BP) into OPF also showed improved mineralization and osteoblast precursor cell attachment, proliferation, and differentiation [20, 22]. Olthof et al. showed that functionalizing OPF hydrogels with 20% *w*/*w* BP could enhance the bone regeneration rate in both ectopic and orthotopic bone defect models, providing a useful approach to improve the biological and mechanical properties of polymer holding vast potential for bone regeneration [20].

In this study, microporous OPF/BP hydrogels were prepared by the salt leaching method. SDF-1*α* was loaded onto microporous OPF/BP hydrogel to obtain SDF-1*α*/OPF/BP composites, and the function of this delivery carrier on rat BMSC migration and osteogenic differentiation was investigated.

## 2. Materials and Methods

### 2.1. Preparation of OPF/BP Hydrogel

OPF was synthesized using poly (ethylene glycol) with an initial molecular weight (Mn) of 1 kDa according to the previously described method [23, 24].

OPF/BP hydrogel was made using the salt leaching method [20]. Firstly, OPF (0.5 g/ml), N-vinyl pyrrolidinone (NVP, 0.15 g/ml; Sigma Aldrich, St. Louis, MO), and Irgacure 2959 (I2959, 0.002 g/ml; Ciba Specialty Chemicals, Tarrytown, NY) were added and mixed in double-distilled water (ddH_2_O). Then, 200 mg BP was added to make OPF/BP paste. The OPF/BP paste (50% *w*/*w*) was mixed with NaCl salt particles (50% *w*/*w*, 100-200 *μ*m) to create the final paste for the composite. The resulting mixture was immediately transferred to 0.8 mm thick silicone rubber molds sandwiched between two glass plates. Then, the mixture was exposed to a UV light (UV-Handleuchte lamp A., Hartestein, Germany) to cross-link for 2 hours. After removal from the molds, hydrogels were punched into disc-shaped specimens using a cork borer (~5 mm in diameter) for further use. The composites were immersed in sterile ddH_2_O to leach out the salt. Then, the microporous OPF/BP hydrogel was freeze-dried by lyophilization. Three samples were sent for scanning electron microscope (SEM) and atomic force microscopy (AFM) examination, respectively.

### 2.2. Characterization of OPF/BP Hydrogel

#### 2.2.1. SEM

The morphology of OPF/BP hydrogels dried by lyophilization was observed using a scanning electron microscope (S-4700, Hitachi Instruments, Tokyo, Japan). For SEM imaging, the surface of the samples was sputter-coated with gold and palladium for 60 seconds and then detected at 3000 V accelerating voltage.

#### 2.2.2. AFM

AFM was utilized to assess the nanoscale morphology of OPF/BP hydrogels using previously published protocols [25]. In brief, the OPF/BP hydrogel was placed onto the top of a fresh-surface mica disc (Ted Pella, Redding, CA). Nanoscale images (512 × 512 pixel resolution) were taken by a Nanoscope IV PicoFroce Multimode AFM (Bruker, Camarillo, CA), using the contact mode at room temperature. The surface roughness (Rq, root mean square) was calculated and averaged from five different areas on the AFM images.

### 2.3. Preparation of SDF-1*α*/OPF/BP Composites

The recombinant murine SDF-1*α* (PeproTech, Rocky Hill, NJ) was loaded onto the microporous OPF/BP by adsorption. Firstly, the SDF-1*α* was diluted in phosphate-buffered saline (PBS) (Gibco, Carlsbad, CA) in concentrations of 0 ng/ml, 250 ng/ml, 500 ng/ml, and 1000 ng/ml. Then, 50 *μ*l SDF-1*α* solutions at different concentrations were pipetted evenly on one side of hydrogels. After 1 hour, other 50 *μ*l SDF-1*α* solutions were pipetted evenly on the other side of the hydrogels. SDF-1*α* was loaded onto the OPF/BP hydrogels to generate four groups of composites with final SDF-1*α* concentrations of 0 ng/ml (control), 50 ng/ml (SDF50), 100 ng/ml (SDF100), or 200 ng/ml (SDF200), according to the culture system of 500 *μ*l. Finally, the SDF-1*α*/OPF/BP composites were dried in the hood for 4 hours prior to further use.

### 2.4. *In Vitro* SDF-1*α* Release

Four samples of SDF-1*α*/OPF/BP composite in each group were placed in a 24-well plate (Corning, Corning, NY) and exposed to 1-, 2-, 3-, 4-, 5-, 14-, and 21-day culture in 500 *μ*l complete medium (CM) (Dulbecco's modified Eagle's medium (DMEM) (Gibco) supplemented with 10% fetal bovine serum (Gibco) and 100 U/ml penicillin-streptomycin (Gibco)). The medium was collected and subjected to ELISA assays of SDF-1*α* (RayBiotech, Peachtree Corners, GA), according to the manufacturer's protocol. The obtained values in the SDF50, SDF100, and SDF200 groups were subtracted by the values of the control group to preclude the preexisting SDF-1*α*. Finally, the concentrations of SDF-1*α* were calculated by the standard curve.

### 2.5. Two-Dimensional Migration Assay

Migration of rat BMSCs (Sprague-Dawley, Fisher Scientific, PA) was assessed by the scratching assay. Three replicates were done for each group. BMSCs were plated in a 24-well plate to create a confluent monolayer. After incubating the dishes properly for approximately 12 h in the incubator with 5% CO_2_ and 95% relative humidity at 37°C, a linear wound midline was made across the bottom using a 200 *μ*l sterile pipet tip. The dish was rinsed gently with phosphate-buffered saline (PBS) to remove any remaining cell debris. SDF-1*α*/OPF/BP composites were added into the corresponding well, and then, 500 *μ*l DMEM was added into each well. Micrographs of the cells were taken using a digital Axiovert 25 Zeiss light microscope (Zeiss, Oberkochen, Germany) at time points 0 h and 48 h. The blank area without cells was quantified by ImageJ software (La Jolla, CA).

C-X-C chemokine receptor type 4 (CXCR4) expressions were assessed by immunofluorescence staining. Three replicates were done for each group. BMSCs were plated on the glass slides in a 24-well plate. SDF-1*α*/OPF/BP composites were added into the corresponding well, and then, 500 *μ*l complete medium was added into each well. At 96 h, BMSCs were fixed with 4% paraformaldehyde (PFA) solution at room temperature for 1 hour and permeabilized by 0.2% Triton X-100 solution at room temperature for another hour. They were then immersed in 3% bovine serum albumin (BSA)/PBS solution at 37°C for 30 minutes to block nonspecific binding sites. The cells were then incubated with anti-CXCR-4 antibody (1 : 100 in PBS, Santa Cruz, sc-53534) at 4°C overnight. This was followed by the incubation with goat anti-rabbit IgG (Alexa Fluor 488, Abcam, ab150077) for 2 hours. Cell nuclei were finally labeled by 4′,6-diamidino-2-phenylindole (DAPI, Sigma-Aldrich, St. Louis, MO) at 37°C for 10 min. The stained cells were immediately imaged. The analysis of fluorescence integral density was done with ImageJ software.

The Transwell assay was performed using chambers with 8 *μ*m pores purchased from Corning. Three replicates were done for each group. BMSCs were harvested and resuspended in 100 *μ*l DMEM at a concentration of 1 × 10^6^ cells and then seeded into the upper chambers of the 24-well plate. SDF-1*α*/OPF/BP composites were placed into lower chambers, and 400 *μ*l DMEM was subsequently added into the lower chambers. Cells were incubated for 48 h in the incubator. Use a cotton-tipped applicator to carefully remove the remaining cells that have not migrated from the top of the membrane without damaging it. Then, the cells that migrated to the reverse side of the Transwell membrane were stained with calcein AM staining kit (Invitrogen, Carlsbad, CA). Micrographs of the cells were taken using a digital Axiovert 25 Zeiss fluorescence microscope at time points 24 h and 48 h. The ImageJ software was utilized to analyze the cell numbers at the reverse side of the Transwell membrane.

### 2.6. Three-Dimensional Migration Assay

**Figure 1:**
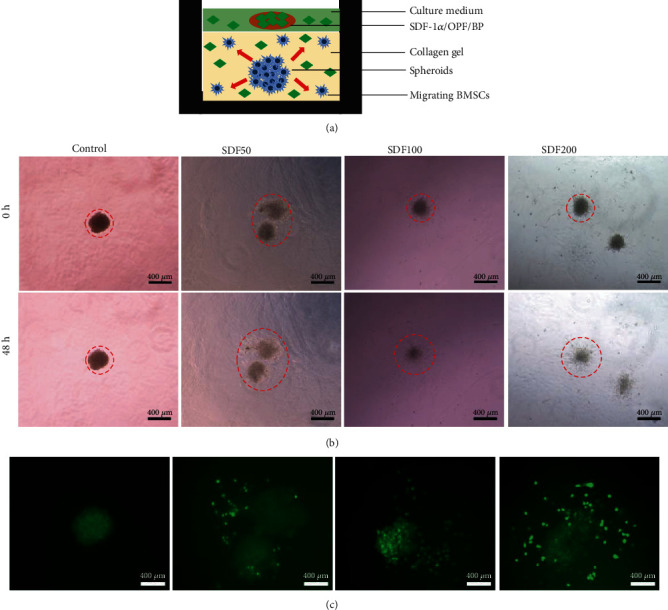
Three-dimensional migration assay of MSCs on the four groups of SDF-1*α*/OPF/BP composites. (a) Schematic illustration of the three-dimensional migration model. (b) 3D spheroid migration assay showed that the migrating distances of the SDF100 and SDF200 groups were higher than those of the SDF50 and control groups. (c) Calcein AM staining of 3D spheroids after 72 hours of culture with SDF-1*α*/OPF/BP composites. The red line indicates the distance of migrating cells.

Three replicates were done for each group. Rat BMSC spheroids were constructed using previously published protocols [26]. Rat BMSCs were trypsinized from the culture dishes and diluted in the complete medium at the concentration of 2 × 10^5^/ml. Then, 50 *μ*l cell solution was added to each well of Corning spheroid microplates to form cell spheroids with individual spheroids consisting of 10^4^ cells. The microplate was centrifuged at 1500 rpm for 5 minutes and then maintained in the incubator. After 24 h, the spheroids were harvested, transferred to a 48-well plate, and suspended in 3 mg/ml collagen gel. After gel cross-linking, SDF-1*α*/OPF/BP composites and 500 *μ*l DMEM were added into each well with spheroids (Figure 1(a)). Three replicates were done for each group. Micrographs were taken at time points 0 and 72 h, and calcein AM staining was performed at 72 h. The migration distance of BMSCs was calculated by the average distance from the core of spheroids.

### 2.7. Osteogenic Assay

#### 2.7.1. Osteogenic Differentiation

Fifteen replicates were done for each group. SDF-1*α*/OPF/BP composites were placed into 24-well plates. Then, BMSCs were plated onto the SDF-1*α*/OPF/BP composites. On day 1, the complete medium was changed with osteogenic medium supplemented with 10 nM dexamethasone (DEX), 10 mM *β*-glycerophosphate sodium, and 50 mg/ml ascorbic acid (AA) (Sigma-Aldrich, St. Louis, MO). The medium was replaced every 3 days. On days 14 and 21, culture medium or cells were collected to detect their osteogenic abilities.

#### 2.7.2. Immunofluorescence (IF) Staining

IF staining were constructed using previously published protocols [26]. On day 21, BMSCs under osteogenic differentiation were fixed with 4% paraformaldehyde (PFA) solution at room temperature for 1 hour and permeabilized by 0.2% Triton X-100 solution at room temperature for another hour. They were then immersed in 3% bovine serum albumin (BSA)/PBS solution at 37°C for 30 minutes to block nonspecific binding sites. The cells were then incubated with anti-RUNX2 antibody (1 : 100 in PBS, Abcam, ab23981) and anti-OPN antibody (1 : 100 in PBS, Abcam, ab8448) at 4°C overnight. This was followed by the incubation with goat anti-rabbit IgG (Alexa Fluor 488, Abcam, ab150077) for 2 hours. Cell nuclei were finally labeled by 4′,6-diamidino-2-phenylindole (DAPI, Sigma-Aldrich, St. Louis, MO) at 37°C for 10 min. The stained cells were immediately imaged using LSM 780 Zeiss Confocal Microscopes (Zeiss, Oberkochen, Germany). The analysis of fluorescence stained area was done with ImageJ software.

#### 2.7.3. Alkaline Phosphatase (ALP) Assay

On days 14 and 21, BMSCs were washed with PBS, trypsinized, and then resuspended in the complete medium. 50 *μ*l cell suspension was used for the calculation of cell numbers with a hemacytometer. The left cell suspension was centrifuged at 1000 rpm and wash with PBS three times. Then, the BMSCs were lysed by 0.2% Triton X-100 solution by shaking for 30 minutes. The ALP concentration was measured using a QuantiChrome™ Alkaline Phosphatase Assay Kit (BioAssay Systems, Hayward, CA). The final ALP concentrations were normalized with the cell number.

#### 2.7.4. Osteocalcin (OCN) Assay

On days 14 and 21, OCN concentration released into the culture medium was quantified utilizing the Rat Osteocalcin Enzyme Immunoassay Kit (Alfa Aesar, Haverhill, MA). Three replicates were done for each group. The final OCN concentrations were normalized with the cell number.

#### 2.7.5. Alizarin Red S Staining

On day 21, BMSCs were fixed with 4% paraformaldehyde (PFA) solution for 1 hour. Then, the wells were washed in ddH_2_O for 10 min before staining followed by the treatment with Alizarin Red S solution (Spectrum, Stamford, CT) for 30 minutes. After washing three times with ddH_2_O, the wells were imaged. The analysis of the stained area was done with ImageJ software.

### 2.8. Statistical Analysis

GraphPad Prism 7 was used for statistical analysis. One-way analysis of variance (ANOVA) was used to determine the differences between the groups, and further, multiple comparisons were conducted using Tukey's honest significant difference test. A *p* value of <0.05 was considered to be significantly different.

## 3. Results

### 3.1. Characteristics of OPF/BP Microporous Hydrogel

**Figure 2:**
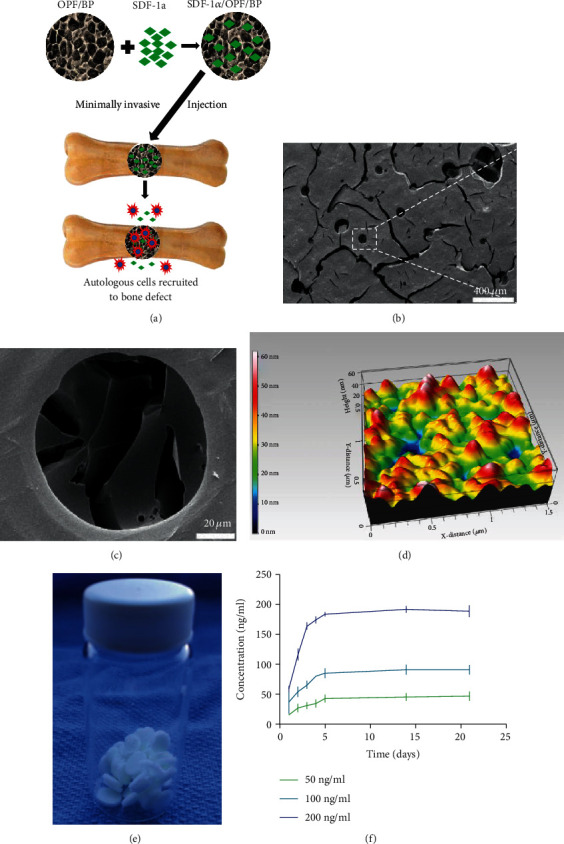
(a) Schematic illustration of the construction of SDF-1*α*/OPF/BP composites for the repair of the bone defect. (b, c) SEM images of OPF/BP hydrogel. (d) AFM image of OPF/BP hydrogel. (e) Photograph of OPF/BP hydrogel discs. (f) Release profiles of SDF-1*α*/OPF/BP composites showed the concentrations of SDF-1*α* in the culture medium. The release curve showed all groups reached 80% release percentage on the 4th day.

SEM images of the OPF/BP hydrogels after salt leaching showed a highly porous interconnected network with micropores randomly distributed in the polymer matrix (Figures 2(b) and 2(c)). The average diameter of the micropores was about 118.3 ± 26.4 *μ*m. AFM scanning showed the average area roughness of OPF/BP hydrogels was 32.2 ± 29.8 nm (Figure 2(d)).

The release profiles of all SDF-1*α*/OPF/BP composites showed an initial burst release (50%) for the first two days. In the SDF200 group, the concentrations of SDF-1*α* released from the composites reached 163 ng/ml (81.5%) on the 3^rd^ day, which leveled off to a more sustained release for the rest of the time course. In the SDF100 group, the concentrations of SDF-1*α* released from the composites reached 80 ng/ml (80%) on the 5^th^ day and then leveled off to a more sustained release. Finally, in the SDF50 group, the concentrations of SDF-1*α* released from the composites reached 42 ng/ml (84%) on the 5^th^ day. In all the groups, the release rate of SDF-1*α* reached about 90% on the 14^th^ and 21^st^ days (Figure 2(f)).

### 3.2. BMSC Chemotaxis to SDF-1*α*/OPF/BP Composites

**Figure 3:**
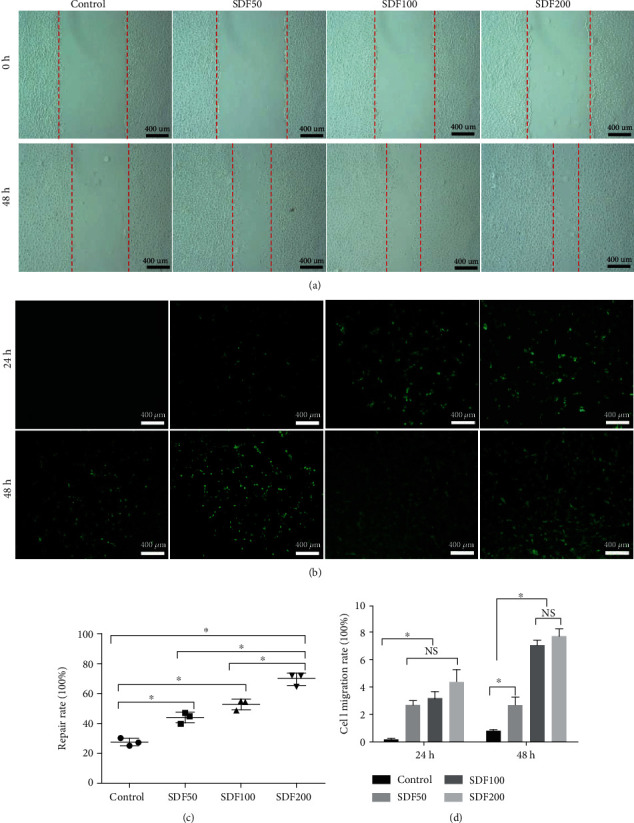
Two-dimensional migration assay of MSCs on the four groups of SDF-1*α*/OPF/BP composites. (a) Scratching assay of MSCs (light micrographs) and (b) Transwell assay of MSCs (fluorescent micrographs stained by calcein AM staining). (c) Scratching assay showed that the wound healing rates of the SDF100 and SDF200 groups were significantly higher (^∗^*p* < 0.05) than those of the SDF50 and control groups. (d) Transwell assay showed that the migrating rates of the three SDF groups were significantly higher (^∗^*p* < 0.05) than those of the control group on the first day, and the migrating rates of the SDF100 and SDF200 groups were significantly higher (^∗^*p* < 0.05) than those of the SDF50 and control groups on the second day. The red line indicates areas without migrating cells.

The presence of SDF-1*α* enhanced the motility and migration of BMSCs in comparison to the control group. Cell migration was elucidated as the filling of the central gap area after making the scratch. During the wound healing assay, the average repair rate of the SDF200, SDF100, SDF50, and control group was 69.7%, 52.7%, 43.8%, and 27.3%, respectively. The cell migration rate of the SDF200 group was significantly higher (*p* < 0.05) than that of the other groups (Figures 3(a) and 3(c)).

Transwell assay showed that the average migration rate of the SDF200, SDF100, SDF50, and control group was 4.4%, 3.2%, 2.7%, and 0.2%, respectively, after 24 hours. After 48 hours, the corresponding average migration rate was 7.8%, 7.1%, 2.7%, and 0.8%. The migration rates of the SDF200 and SDF100 groups were significantly higher (*p* < 0.05) than those of the SDF50 and control groups (Figures 3(b) and 3(d)).

The 3D migration assay showed that in the SDF200 and SDF100 groups, the border of spheroids extended more than 1.5 folds, while the SDF50 group extended less than 1.2 folds, and the control group almost stayed the same (Figures 1(b) and 1(c)), indicating the enhanced chemotaxis effect of SDF at higher concentrations.

**Figure 4:**
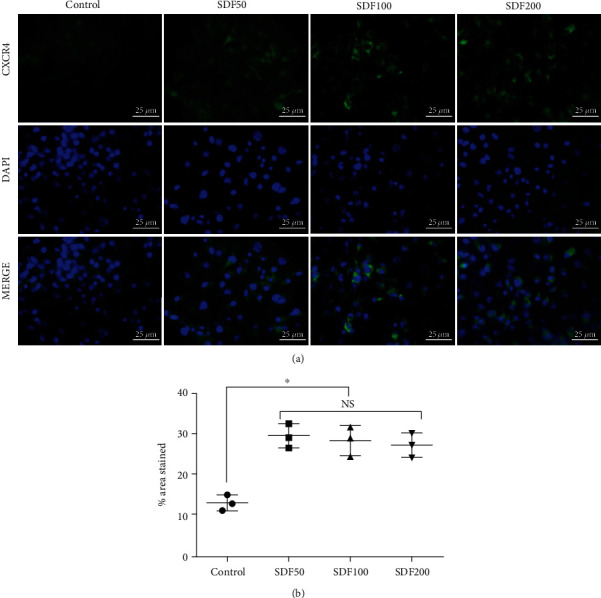
CXCR4 immunofluorescence staining of rat BMSCs. (a) CXCR4 immunofluorescence staining after 96 hours of coculture with SDF-1*α*/OPF/BP composites. (b) The percentage of CXCR4-positive areas of the SDF200, SDF100, and SDF50 groups was significantly higher (^∗^*p* < 0.05) than that of the control group.

SDF-1*α* enhanced the expression levels of CXCR-4 in BMSCs. The expression levels of CXCR-4 in the SDF50, SDF200, and SDF100 groups were significantly higher (*p* < 0.05) than those of the control group, but there was no statistical difference between SDF50, SDF200, and SDF100 groups (*p* > 0.05) (Figure 4).

### 3.3. BMSC Osteogenic Differentiation on SDF-1*α*/OPF/BP Composites

**Figure 5:**
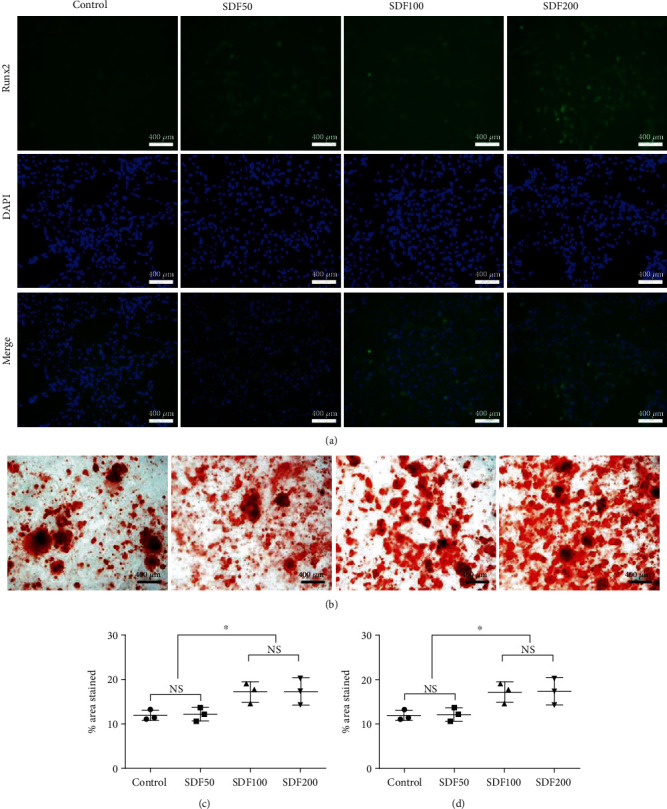
Osteogenic differentiation assays of MSCs after 21 days of culture on the four groups of SDF-1*α*/OPF/BP composites. (a) Runx2 immunofluorescence staining on the 21^st^ day of osteogenic differentiation. (b) Alizarin Red S staining on the 21^st^ day of osteogenic differentiation. (c) The percentage of Runx2-positive areas of the SDF200 and SDF100 groups was significantly higher (^∗^*p* < 0.05) than that of the SDF50 and control groups. (d) The percentage of Alizarin Red S stained area of the SDF200 and SDF100 groups was significantly higher (^∗^*p* < 0.05) than that of the SDF50 and control groups.

**Figure 6:**
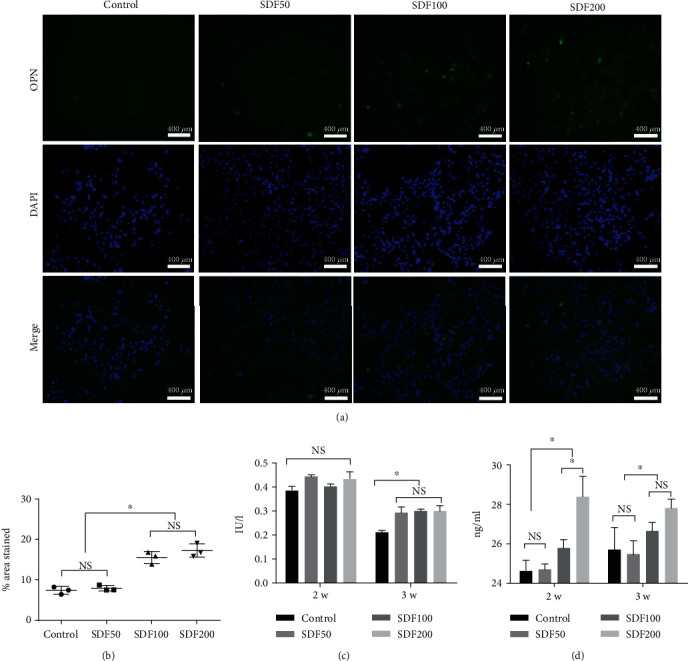
Osteogenic differentiation assays of MSCs after 14 and 21 days of culture on the four groups of SDF-1*α*/OPF/BP composites. (a) OPN immunofluorescence staining on the 21^st^ day of osteogenic differentiation. (b) The percentage of OPN-positive areas of the SDF200 and SDF100 groups was significantly higher (^∗^*p* < 0.05) than that of the SDF50 and control groups. (c) ALP assay showed that ALP expression levels of the four groups were similar at day 14, while the expression levels in the control group were significantly lower (^∗^*p* < 0.05) than those of the other three groups on the 21^st^ day. (d) OCN assay showed that the OCN expression levels in the SDF200 and SDF100 groups were significantly higher (^∗^*p* < 0.05) than those of the SDF50 and control groups on both days 14 and 21.

After 21 days of osteogenic differentiation, BMSCs in all the groups expressed Runx2 and OPN. The expression levels of Runx2 and OPN in the SDF200 and SDF100 groups were significantly higher (*p* < 0.05) than those of the SDF50 and control groups, and there was no statistical difference between the SDF200 and SDF100 groups (*p* > 0.05) (Figures 5(a) and 5(c) and 6(a) and 6(b)). Alizarin Red S staining also confirmed that the SDF200 and SDF100 groups had higher mineral deposition than that of the SDF50 and control groups (*p* < 0.05), and there was no statistical difference between the SDF200 and SDF100 groups (*p* > 0.05) (Figures 5(b) and 5(d)).

On the 14^th^ day, the expression levels of ALP showed no difference among the four groups, while its expression levels on the 21^st^ day in the SDF200, SDF100, and SDF50 groups were higher than those of the control group with statistical significance (*p* < 0.05), and there was no statistical difference between the SDF50, SDF200, and SDF100 groups (*p* > 0.05) (Figure 6(c)). The expression levels of OCN in the SDF200 and SDF100 groups were also significantly higher (*p* < 0.05) than those of the SDF50 and control groups both on the 14^th^ and 21^st^ days. On the 14^th^ day, the expression levels of OCN in the SDF200 group were also significantly higher than those of the SDF100 group, and on the 21^st^ day, there was no statistical difference between the SDF200 and SDF100 groups (*p* > 0.05) (Figure 6(d)).

## 4. Discussion

In this study, we made SDF-1*α*/OPF/BP composites and investigated their potential on the migrating and osteogenic abilities of rat BMSCs. We found that SDF-1*α* released from the composites significantly promoted the homing and osteogenic abilities of BMSCs, and the optimum concentration of SDF-1*α* was 100 ng/ml.

Tissue engineering techniques dependent on stem cell transplantation are widely used in bone regeneration [26]. The drawbacks of the cell transplantation-based methods such as significant expense, time-wasting, potential infection, and low rates of cell survival (2-4%) limit their further clinical application [2, 10]. The cell guidance method, which motivates the migration and homing of stem cells, is an alternative approach. Thus, autologous MSC guidance is being considered as a feasible alternative treatment method as it does not require massive *in vitro* cell proliferation. Recently, a lot of chemokines or chemotactic drugs have been proved to possess the ability to recruit autologous cells to facilitate tissue regeneration [27, 28].

One promising factor in promoting cell migration is SDF-1*α* [29], a ligand of C-X-C chemokine receptor type 4 (CXCR4) expressed on the surface of many types of stem cells or progenitor cells [12]. SDF-1*α* plays a vital role in the recruitment of stem cells and also participates in the differentiation process of BMSCs, which is directly related to its ability to bind CXCR4 [12, 19]. However, there is a temporal mismatch between the peak expression of SDF-1*α* and the upregulation of CXCR4 [13]. Within an hour of injury, SDF-1*α* expression is rapidly upregulated, whereas CXCR4 peaks at 96 hours [30–32].

Previous studies have used OPF/BP microporous hydrogel as the carrier for BPM-2 (13 kDa), which has a similar molecular mass as SDF-1*α* (10 kDa), and showed that BMP-2 burst release (80% within 4 days) from OPF/BP composites produced more bone compared to other sustained release systems [22]. In this study, SDF-1*α* also showed a burst release from the OPF/BP hydrogel (80% within 4 days), which matched the upregulation of CXCR4 on stem cells *in vivo*. We also found that the SDF-1*α* could significantly enhance the expression of CXCR4 in rat BMSCs *in vitro*. This study showed that the burst release of SDF-1*α* could significantly improve the migration abilities of MSCs either in 2D migration assays or 3D spheroid assays. The migration abilities of MSCs were SDF-1*α* dose-dependent. The concentration of SDF-1*α* beyond 100 ng/ml, however, showed nonsignificant effects on the migration ability of MSCs. Previous studies also showed similar findings related to the optimum concentration of SDF-1*α* [14, 30].

OPF/BP can also provide a proper framework to achieve appropriate bone formation. The phosphates in the OPF-BP matrix contributed to the enhanced osteogenic activities such as attachment, proliferation of MSCs and osteoblasts, and tissue mineralization [20, 22]. Furthermore, SDF-1*α* is also known for its ability to promote the osteogenic differentiation of MSCs [17]. Thus, in this study, the composites developed combining OPF/BP with SDF-1*α* were able to induce increased mineral deposition and a significant increase in the expression levels of Runx2, OCN, OPN, and ALP—four vital osteogenic proteins representing both early and late stages of bone regeneration—to promote the osteogenic abilities of MSCs.

## 5. Conclusion

In conclusion, microporous SDF-1*α*/OP/BP composite promoted the migration and osteogenic differentiation of MSCs and thus represented a promising candidate material for bone tissue regeneration. The optimized concentration of SDF-1*α* for enhanced activities was determined to be 100 ng/ml in our study.

## Data Availability

The data used to support the findings of this study are available from the corresponding author upon request.
